# *Ganoderma lingzhi* Triterpenoids Ameliorate CCl_4_-Induced Acute Liver Injury in Mice

**DOI:** 10.3390/foods15101662

**Published:** 2026-05-10

**Authors:** Shuran Wen, Zhanshan Ma, Dongling Zhan

**Affiliations:** College of Food Science and Engineering, Jilin Agricultural University, 2888 Xincheng Street, Changchun 130118, China; wenshuran2023@163.com (S.W.); 18604493504@163.com (Z.M.)

**Keywords:** *Ganoderma lingzhi* triterpenoids, acute liver injury, protective mechanism, anti-inflammatory effect, antioxidant effect, Nrf2/Keap1, MyD88/NF-kB-p65

## Abstract

The liver is a critical organ in drug metabolism and detoxification. *Ganoderma lingzhi* triterpenoids, a major class of bioactive compounds in *G. lingzhi* extracts, exhibit liver protective effects with pharmaceutical potential. In this study, we established an acute liver injury model in mice via intraperitoneal injection of 0.25% Carbon tetrachloride(CCl_4_) olive oil. Prophylactic and therapeutic administration of *G. lingzhi* triterpenoid extracts were evaluated using alanine aminotransferase (ALT), aspartate aminotransferase (AST), superoxide dismutase (SOD), malondialdehyde (MDA), glutathione peroxidase (GSH-Px), tumor necrosis factor-α (TNF-α), interleukin-6 (IL-6), and hepatic histopathology. Western blot analysis assessed protein expression of anti-inflammatory and antioxidant stress-related pathways (Nrf2/Keap1 and MyD88/NF-κB-p65). Intervention effects on acute liver injury were determined by measuring protein molecular weight following triterpenoid treatment. In summary, *G. lingzhi* triterpenoids significantly alleviate oxidative stress and inflammatory responses in mice with acute liver injury by activating the KEAP1-Nrf2 antioxidant pathway and inhibiting the NF-κB-p65 and MyD88-mediated inflammatory pathways. These triterpenoids reduced serum transaminase levels, improved hepatic histopathological damage, and exerted effective protective effect on liver tissue. This study provides experimental support for the comprehensive evaluation of *G. lingzhi*’s anti-inflammatory and antioxidant effects.

## 1. Introduction

**Figure 1:**
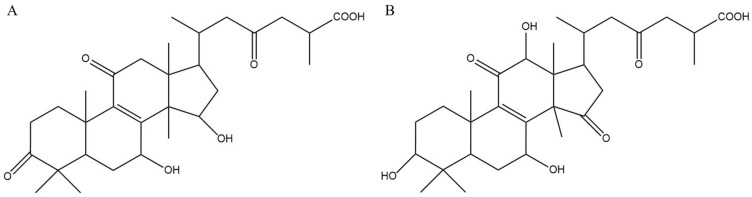
Structures of ganoderic acid A (**A**) and ganoderic acid G (**B**).

As a distinctive traditional Chinese medicine, *Ganoderma lingzhi* was first documented in the ancient pharmacopoeia Shennong Ben Cao Jing and possesses various medicinal properties. *G. lingzhi* is a prominent medicinal fungus, and its active constituents include triterpenoids, polysaccharides, sterols, and alkaloids [1]. It can enhance the immune system of diabetic patients and alleviate symptoms of the condition [2]. Current reports on *G. lingzhi* bioactivities primarily focus on its cytotoxic, antitumor, antioxidant, hepatoprotective, neuroprotective, and antidiabetic properties [3]. Among these, *G. lingzhi* triterpenoids, which are extensively oxidized derivatives of lanosterane, are significant active components in the extract and are promising for pharmacological and medical applications [4,5,6]. The triterpenoid content in *G. lingzhi* has become a quality evaluation criterion in the United States Pharmacopeia [7]. Recent pharmacological studies have demonstrated that *G. lingzhi* triterpenoids possess diverse biological activities, including antitumor, antibacterial, antiviral, immunomodulatory, antioxidant, and hepatoprotective effects, as well as glucose and lipid regulation [8,9,10,11,12]. As shown in Figure 1, *G. lingzhi* triterpenoids, as highly oxidized lanostane derivatives, are classified according to their functional groups, including ganoderic acids, alcohols, aldehydes, and lactones [13]. Based on structural characteristics, Jiang et al. evaluated ganoderic acids with different structures and their varying inhibitory effects on breast cancer cells (MDA-MB-231), and they determined that hydroxylation at the C-7, C-15, and C-3 positions on the triterpenoid ring exerted the strongest suppression of malignant cell growth and metastasis [14].

The liver is one of the major organs involved in drug metabolism and detoxification processes [15]. However, drugs or their metabolites may induce hepatotoxicity. CCl_4_ is a well-established hepatotoxin metabolized in the liver to generate reactive free radicals that induce hepatocyte injury and necrosis [16]. The MyD88/NF-κB signaling pathway mediates inflammatory responses during liver injury [17,18]. Activation of this pathway upregulates pro-inflammatory cytokines such as TNF-α and IL-6, thereby aggravating hepatic damage [19]. Targeting the MyD88/NF-κB axis has been demonstrated to attenuate inflammation and protect against liver injury [20].

Multiple studies have demonstrated the efficacy of *G. lingzhi* in attenuating multi-organ inflammation, particularly in the spleen and liver [21,22]. Ma reported that ganoderic acid A could effectively inhibit hepatocyte apoptosis by suppressing the expression of proteins in the toll-like receptor/nuclear factor-κB (TLR/NF-κB) and apoptosis signaling pathways [23]. Therefore, we hypothesized that dibutyl phthalate (DBP)- and benzo[a]pyrene (BaP)-induced liver injury in rats might be mediated by the activation of TLR4ac and MyD88/NF-κB signaling pathways. Gao et al. divided 30 patients with HBsAg-positive chronic active hepatitis into treatment and control groups [24]. The treatment group received *G. lingzhi* slices without concurrent hepatoprotective medications, while the control group received *Schisandra chinensis* seed oil. Following a 30-day treatment period, patients were evaluated. Results indicated that the overall efficacy rate in treating HBsAg-positive chronic active hepatitis was significantly greater in the experimental group compared to the control group. Therefore, this study aimed to investigate the roles and potential mechanisms of the TLR4ac and MyD88/NF-κB signaling pathways in DBP- and BaP-induced liver injury by establishing single- and co-exposure rat models and to identify key toxic effect nodes to facilitate future therapeutic research [25].

## 2. Materials and Methods

### 2.1. Ganoderma Lingzhi Triterpenoids

The *G. lingzhi* spore powder was purchased by this laboratory from the Changchun Specialties Store and stored at the Mycological Herbarium of Jilin Agricultural University. Triterpenoids were extracted and purified from *Ganoderma lingzhi* spore powder following an established protocol [26]. *G. lingzhi* spore powder was processed with 70% ethanol (Beijing Chemical Works, Beijing, China) as the extraction solvent, employing cellulase hydrolysis (Shandong Lonct Enzymes Co., Ltd., Linyi City, China) and ultrasonic-assisted extraction (Shanghai Guante Ultrasonic Instruments Co., Ltd., Shanghai, China). The liquid-to-solid ratio was maintained at 50:1. Hydrolysis temperature at 50 °C for 35 min, followed by ultrasonication for 46 min with an enzyme loading of 4.8%. These conditions yielded a triterpene extract with a concentration of 0.98 mg/g. The *G. lingzhi* triterpenoid extract was purified using AB-8 macroporous resin (Tianjin Tiantai Chemical Co., Ltd., Tianjin, China). The extract was loaded at a concentration of 25 mg/g and a flow rate of 1.0 mL/min. Following 3 h of static adsorption, impurities were removed by washing with distilled water. Elution was performed with 95% ethanol as a flow rate of 1.0 mL/min. After purification with macroporous resin, the purity of the *G. lingzhi* triterpenoids was 87.09%.

High performance liquid chromatography (HPLC) was employed to characterize the *G. lingzhi* triterpenoid extracts (Waters Corporation, Milford, MA, USA). Chromatography was performed on a column (4.6 mm × 250 mm, 5 μm) with gradient elution using acetonitrile (A) and 0.01% acetic acid in water (B) as the mobile phase. Optimal conditions were column temperature 30 °C, flow rate 1.0 mL/min, detection wavelength 254 nm, and injection volume 20 μL. The gradient elution program is presented in Table 1. Individual triterpenoid standard solutions and mixed standard solutions were injected and analyzed separately. Qualitative identification was conducted based on the retention times of triterpenoid components.

**Figure 2:**
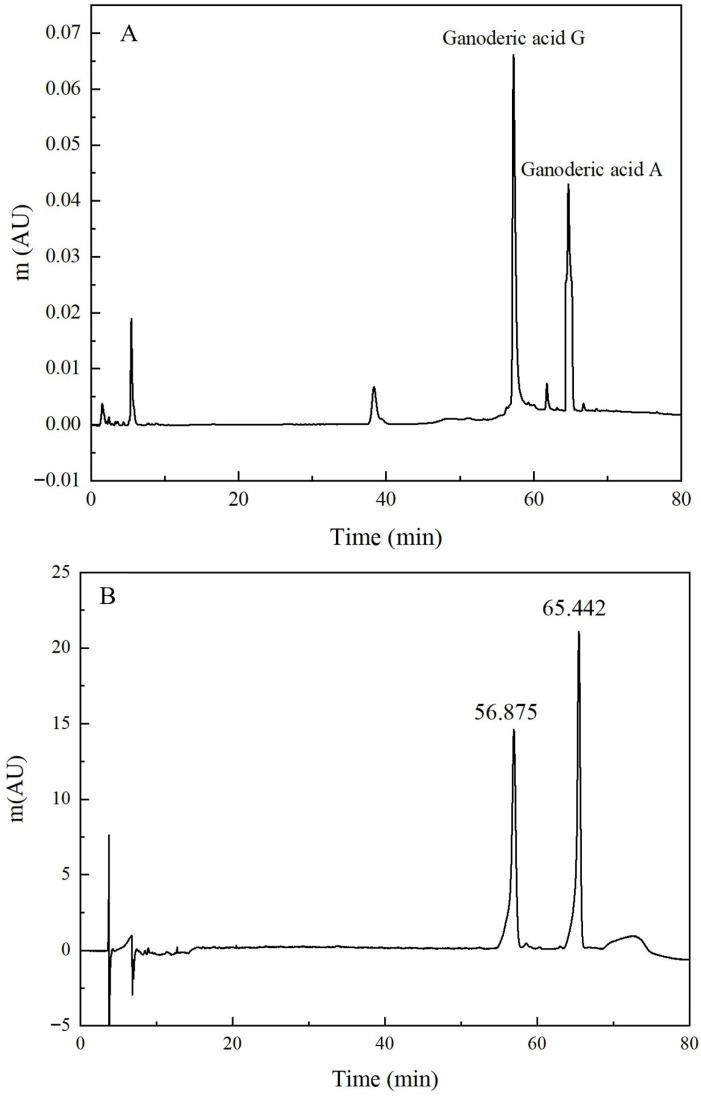
HPLC of mixed components of *G. lingzhi* triterpenoids standard (**A**) and *G. lingzhi* triterpenoids extracts (**B**) Adapted from [26]. (**A**): mixed components of *G. lingzhi* triterpenoids standard; (**B**): *G. lingzhi* triterpenoids extracts.

HPLC chromatograms of the mixed *G. lingzhi* triterpenoid standards and samples are presented in Figure 2. The retention times of the triterpenoid standards were: ganoderic acid G, 56.875 min; and ganoderic acid A, 65.442 min. Calibration curves were constructed by HPLC analysis, yielding the following linear regression equation:

Ganoderic acid A: y = 2.0393 × 10^6^ x – 62,245.5303, R^2^ = 0.9992

Ganoderic acid G: y = 3.8941 × 10^6^ x – 61,808.1393, R^2^ = 0.9994

The results indicated that ganoderic acids exhibited excellent linearity within the concentration range of 0.01–0.1 mg/L. Quantitative analysis revealed that ganoderic acid A and ganoderic acid G comprised 26.17% and 38.35% of the total ganoderic acids, respectively.

### 2.2. Animals

Specific pathogen-free (SPF) Kunming mice were purchased from Beijing VTLH Laboratory Animal Technology Co., Ltd. (Beijing, China). Equal numbers of male and female mice were used (weight, 18–22 g; age, 6–8). Animals were acclimatized for 7 days prior to experimental procedures. Animals were housed under controlled environmental conditions: temperature 22 ± 1 °C, humidity 50 ± 10%, and a 12 h light/dark cycle (Laboratory Animal Center of Jilin Agricultural University; Approval Code: 20221017001). Mice were randomly divided into six groups (*n* = 8 per group): model (LM), blank (LK), biphenyldiphenol-positive (LY), high-dose (LGTG), medium-dose (LGTZ), and low-dose (LGTD). The LGTD, LGTZ, and LGTG groups were administered *G. lingzhi* triterpenoids extracts at doses of 150, 300, and 600 mg/kg/d, respectively [27], via oral gavage once daily for 7 d. The blank and model groups received distilled water, whereas the positive control group received bifendate (Shanghai Macklin Biochemical Co., Ltd., Shanghai, China) at 150 mg/kg/day. On day 6, following drug administration, mice were fasted for 12 h with free access to water, and body weights were recorded. On day 7, 1 h after drug administration, the blank group received physiological saline injection, while other groups received an intraperitoneal injection of 0.25% CCl_4_ (Beijing Chemical Works, Beijing, China) olive oil to induce acute liver injury. Subsequently, at 3 and 12 h post-modeling, mice were administered the corresponding treatments via oral gavage and sacrificed by cervical dislocation 24 h after modeling [28].

### 2.3. Sample Collection and Processing

Blood samples were allowed to clot for 0.5 h at room temperature (approximately 22 °C), then centrifuged at 3500× *g* for 10 min (Agilent Technologies, Inc., Santa Clara, CA, USA) to obtain serum, which was stored at −80 °C (AUCMA COMPANY LIMITED, Qingdao, China). Following cervical dislocation, hepatic tissues were harvested from the mice. The liver tissue was rinsed with physiological saline, blotted dry on filter paper, and weighed. Liver morphology, including size and color, was visually examined. The left and right lobes were immersed in tissue-fixative solution for histopathological analysis.

### 2.4. Determination of Various Indices in Mice

#### 2.4.1. Determination of Body Weight and Organ Indices

The livers were rinsed with saline, dried using filter paper, weighed, and the liver coefficient was determined.

$$\mathrm{Liver}\;\mathrm{Index}=\frac{Liver\;weight\left(g\right)}{\mathrm{Body}\;\mathrm{weight}\;\mathrm{of}\;\mathrm{mice}\left(g\right)}\;\times \;100\%$$

#### 2.4.2. Determination of Biochemical Parameters in the Mouse Serum

Low-temperature thawing was performed on the serum kept at −80 °C. ALT and AST activities were measured according to the instructions of the ALT and AST Biochemical Kit of Nanjing Jiancheng Institute of Biological Engineering (Nanjing, China).

#### 2.4.3. Determination of Liver Tissue-Related Indices

Liver tissue was homogenized in physiological saline at a ratio of 1:9 (*w*/*v*) using an ultra-turrax homogenizer (Shanghai Jingxin Industrial Development Co., Ltd., Shanghai, China), followed by centrifugation at 3500× *g* for 10 min. The supernatant was collected and used for subsequent content determination. The MDA, SOD catalase from *Micrococcus lysodeikticus* (CAT), and GSH-Px levels in the hepatic tissues of all groups of mice were quantified according to the guidelines provided by the biochemical kit (Beijing Solarbio Science & Technology Co., Ltd., Beijing, China). IL-6 and TNF-α levels in mouse hepatic tissue were measured using an enzyme-linked immunosorbent assay kit (ELISA Test Kit, Shanghai Enzyme-linked Biotechnology Co., Ltd., Shanghai, China), following the manufacturer’s instructions.

### 2.5. Observation of Tissue Section and Pathology

Before fixation, liver tissues were rinsed thoroughly with physiological saline and immersed in 4% paraformaldehyde solution (Jilin Jiahang Biotechnology Co., Ltd., Changchun, China) for histopathological examination.

### 2.6. Assays of Inflammation- and Antioxidant-Related Proteins

Bifendate (150 mg/kg/day, positive control) was administered to the LY group as described in Section 2.2 as an in vivo anti-inflammatory and antioxidant reference. Inflammation- and antioxidant-related proteins in liver tissues were analyzed using Western blotting. Liver tissue was washed with ice-cold PBS and homogenized with tissue protein extraction reagent. Following tissue lysis, the homogenate was centrifuged at 12,000× *g* for 15 min at 4 °C, and the supernatant was collected for protein quantification [29]. Western blotting was performed as follows: the addition of 5× loading buffer to each sample, denaturing of the proteins in a boiling water bath, gel preparation, gel filling, sample loading, electrophoresis initiation, stopping of the electrophoresis when the target proteins ran to the appropriate position, membrane transfer, membrane blocking, primary antibody incubation and cultivation, secondary antibody incubation and cultivation, sample washing with Tris-buffered saline with tween (TBST, Beijing Solarbio Science & Technology Co., Ltd., Beijing, China), the addition of enhanced chemiluminescence (ECL, Bio-Rad Laboratories, Hercules, CA, USA) mixed solution dropwise, detection, and analysis of the optical density of the target bands.

### 2.7. Data Processing

Microsoft Excel 2019 and Originpro 8.5 were used for graphing and SPSS 26 was used for data analysis. Data representation was repeated thrice, averaged, and analyzed for significant differences.

## 3. Results

### 3.1. Determination of Body Weight and Liver Index

The liver indexes of the LM group were significantly different from those of the LK group (Table 2), indicating the successful establishment of the acute liver injury model. CCl_4_ caused liver lesions, hypertrophy, and damage in the LM group. The other dose groups showed a significant decrease compared to the LM group, indicating that the administration of *G. lingzhi* triterpenoids alleviated liver tissue damage.

### 3.2. Measurement of Liver Injury Indicators

**Figure 3:**
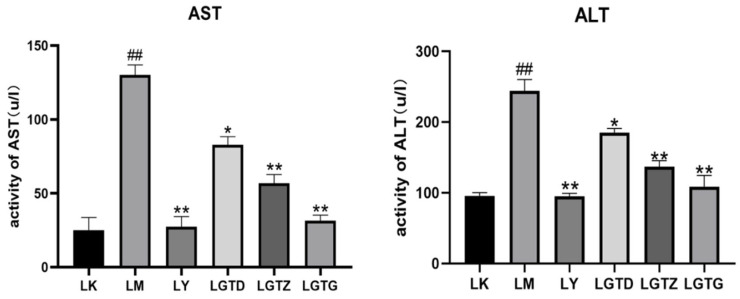
*Ganoderma lingzhi* triterpenoids affect the AST and ALT levels in mouse serum. Note: * *p* < 0.05, ** *p* < 0.01 compared with the LM group, ## *p* < 0.01 compared with the control group.

Serum AST and ALT levels are the primary indicators of hepatic injury severity. The results showed that the blood ALT and AST levels in the LM group were significantly elevated compared with those of the LK group (Figure 3), indicating that the intraperitoneal injection of CCl_4_ induced hepatic cell injury in mice. The ALT and AST levels in the LGTD and LY groups were significantly lower than those in the LM group. Similarly, ALT and AST activities in the LGTZ and LGTG groups were significantly lower than those in the LM group. The AST and ALT activities in the LM group differed significantly from those in the LK group. The AST and ALT activities in the LY, LGTD, LGTZ, and LGTG groups exhibited varying levels of enhancement compared to those in the LM group. Furthermore, the amelioration of liver damage was more significant in the LGTZ and LGTG groups.

### 3.3. Determination of Antioxidant Indicators

**Figure 4:**
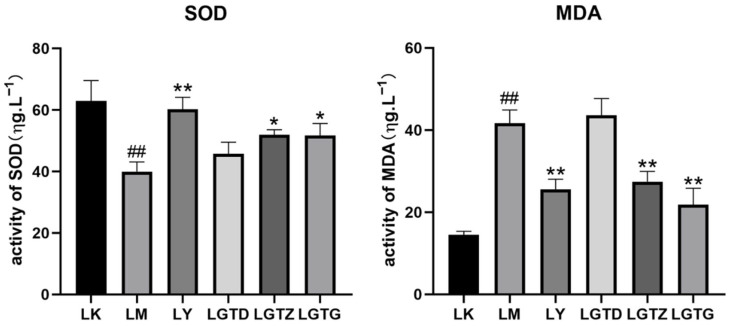

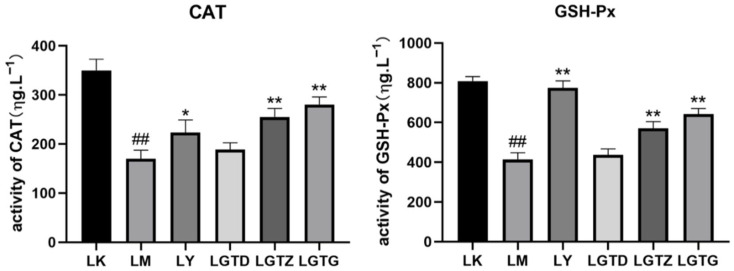
*Ganoderma lingzhi* triterpenoids affect the SOD, MDA, CAT, and GSH-Px levels in the mouse serum. Note: * *p* < 0.05, ** *p* < 0.01 compared with the LM group, ## *p* < 0.01 compared with the control group.

The serum MDA levels and SOD activity are shown in Figure 4. Serum MDA activity in the LM group was significantly higher than that in the LK group, indicating that CCl_4_ induced oxidative stress and altered the serum MDA levels. Serum MDA levels induced by the intraperitoneal injection of CCl_4_ in the LY group were significantly lower than those in the LM group. Significantly lower MDA levels were detected in the LGTZ and LGTG groups than in the LM group. The LGTD diet had a significant effect on MDA levels compared to the LM diet.

Serum SOD activity in the LM group was significantly lower than that in the LK group (Figure 4), indicating that the oxidative stress response was closely related to serum SOD levels. Significantly higher SOD activity was observed in the serum of mice in the LY group than that in the LM group. SOD activity was significantly increased in the serum of mice in the LGTZ and LGTG groups. However, the reduced serum SOD activity in the LGTD group did not improve the acute liver injury.

For CAT, the LY, LGTZ, and LGTG groups showed significantly greater activity than the LM group (Figure 4). In the LGTZ and LGTG groups, the activity of LGTZ and LGTG groups increased significantly, and the activity of LY, LGTZ, and LGTG groups was significant compared to that of the LM group in the GSH-Px indexes analysis. The activities of the LGTZ and LGTG groups were significantly increased. However, no significant differences were noted in the CAT and GSH-Px indices between the LGTD and LM groups, indicating that LGTD only slightly improved acute hepatic damage.

### 3.4. Measurement of Inflammatory Indicators

**Figure 5:**
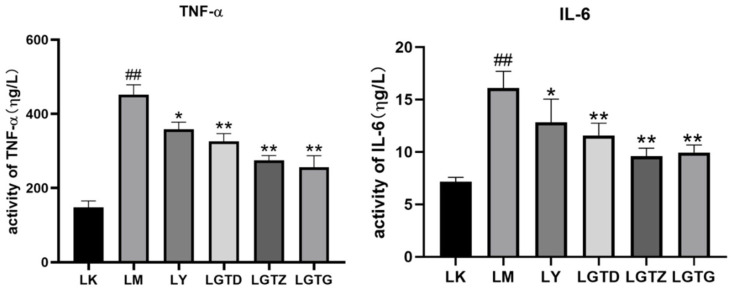
*Ganoderma lingzhi* triterpenoids affect TNF-α and IL-6 levels in the mouse serum. Note: * *p* < 0.05, ** *p* < 0.01 compared with the LM group, ## *p* < 0.01 compared with the control group.

TNF-α and IL-6 are crucial inflammatory factors that regulate inflammatory processes. The hepatic tissues of the mice in the LM group had significantly elevated TNF-α and IL-6 levels compared to those in the LK group (Figure 5). The LGTD, LGTZ, and LGTG group mice exhibited significantly decreased serum TNF-α and IL-6 levels compared to those of the LM group. The increased serum TNF-α and IL-6 levels induced by the intraperitoneal injection of CCl_4_ were significantly decreased in the LY group compared to those in the LM group. In the LGTD, LGTZ, and LGTG groups, serum TNF-α levels were significantly lower than those in the LM group. Similarly, the serum IL-6 levels in the LGTD, LGTZ, and LGTG groups were significantly lower than those in the LM group. Overall, *G. lingzhi* triterpenoids significantly inhibited the activity of inflammatory factors, thereby reducing hepatocyte damage.

### 3.5. Pathological Observation of Tissues

**Figure 6:**
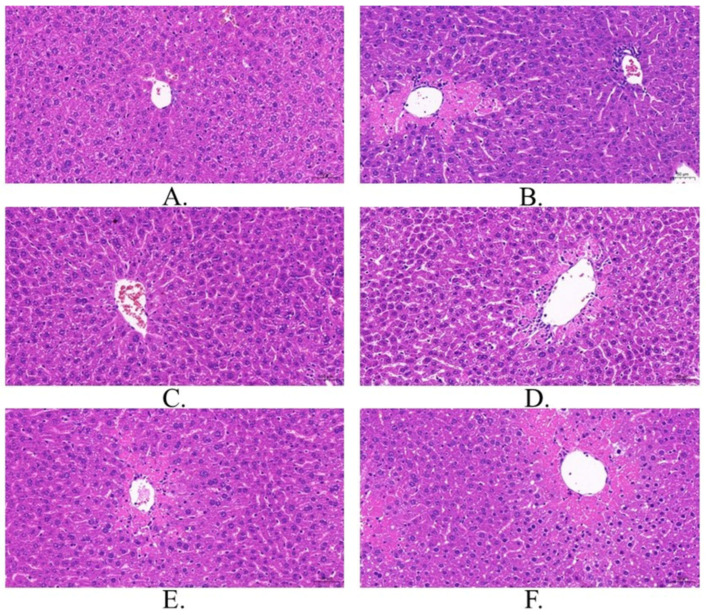
Histopathology of the liver in each mouse group. (**A**): LK group; (**B**): LM group; (**C**): LY group (150 mg/kg); (**D**): LGTD group (150 mg/kg); (**E**): LGTZ group (300 mg/kg); (**F**): LGTG group (600 mg/kg).

Hematoxylin and eosin (HE) staining showed that the hepatic lobules of LK mice were structurally intact, hepatic cords were neatly arranged, hepatocyte morphology was normal with no evident edema or necrosis, and no inflammatory cell infiltration was observed in the confluent area (Figure 6). Compared with observations in the LK mice, LM mice had an incomplete hepatic lobule structure, disorganized hepatic cords, necrosis in the center of the hepatocyte lobules, nuclear consolidation and densification of the nuclei, and inflammatory cell infiltration. Sinusoidal congestion and Kupffer cell hypertrophy were the most common lesions in this group. Vacuolar degeneration of hepatocytes was observed. In the LY group, the structure of the liver lobules was intact, hepatic cords were neatly arranged, hepatocyte structure was intact, and inflammatory cell infiltration and vacuolar degeneration were significantly reduced. A few hepatocytes exhibited necrosis, and inflammatory cell infiltration was minimal in the confluent regions. In the LGTG group, the hepatic lobules and cords were well-arranged, and the structure of the confluent area was normal. In the LGTD and LGTZ groups, the liver lobule structures were disrupted compared to those in the LGTG group, and hepatocellular necrosis and inflammatory cell infiltration were observed. Compared with those of the LM group mice, the liver tissues of the LGTD and LGTZ group mice showed somewhat reduced liver tissue lesions, inflammatory cell infiltration, and vesicular degeneration; however, the improvements were less notable than those exhibited by the LGTG group.

### 3.6. Effects on Liver Tissue-Related Pathways in Mice with Acute Liver Injury

#### 3.6.1. Effects of *G. lingzhi* Triterpenoids on the Protein Expression of Antioxidant Pathway-Related Factors in Mouse Liver Tissues

**Figure 7:**
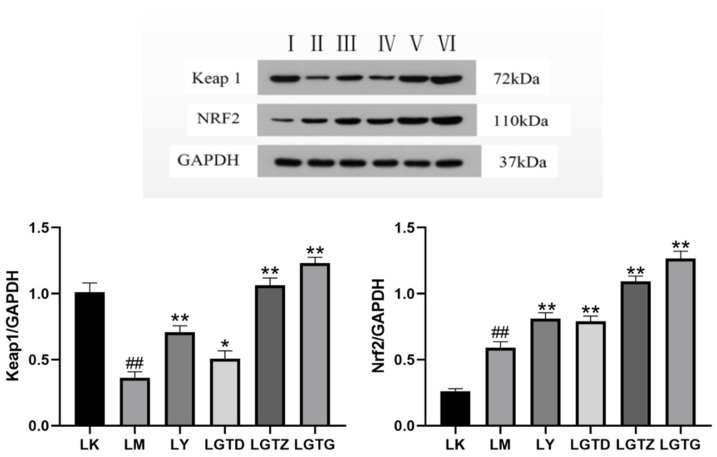
Expression of Keap1 and Nrf2 proteins. I: LK group; II: LM group; III: LY group (150 mg/kg); IV: LGTD group (150 mg/kg); V: LGTZ group (300 mg/kg); VI: LGTG group (600 mg/kg) Note: * *p* < 0.05, ** *p* < 0.01 compared with the LM group, ## *p* < 0.01 compared with the control group.

Western blotting analyses showed that Keap1 expression was significantly lower and that of Nrf2 was significantly higher in the LM group than in the LK group, indicating that acute liver injury triggered an endogenous antioxidant response via the Keap1/Nrf2 pathway (Figure 7). When compared to the LM group, the LGTD, LGTZ, and LGTG groups showed significantly higher Keap1 expression and significantly lower total Nrf2 expression. This seemingly counterintuitive result may be explained by the following: *G. lingzhi* triterpenoids alleviate oxidative stress through direct radical-scavenging activity or by upregulating downstream antioxidant enzymes (e.g., HO-1, NQO1), thereby reducing the compensatory demand for Nrf2 protein synthesis. Moreover, a decrease in total Nrf2 does not preclude enhanced nuclear translocation and transcriptional activity; further immunofluorescence or nuclear fractionation assays would be needed to confirm this. Collectively, these data support that *G. lingzhi* triterpenoids modulate, rather than simply upregulate, the Keap1/Nrf2 axis to restore redox homeostasis.

#### 3.6.2. *G. lingzhi* Triterpenoids Affect the Protein Expression of Inflammatory Pathway-Related Factors in Mouse Liver Tissues

**Figure 8:**
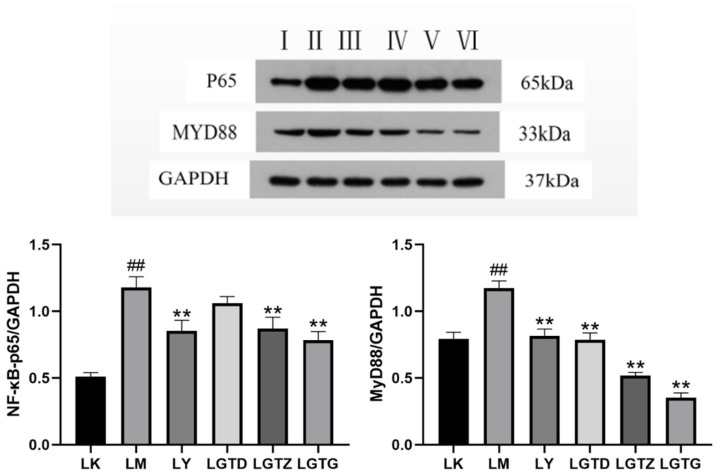
NF-κB-p65 and MyD88 protein expression. I: LK group; II: LM group; III: LY group (150 mg/kg); IV: LGTD group (150 mg/kg); V: LGTZ group (300 mg/kg); VI: LGTG group (600 mg/kg) Note: ** *p* < 0.01 compared with the LM group, ## *p* < 0.01 compared with the control group.

The expression levels of NF-κB-p65 and MyD88 were significantly elevated in the livers of mice in the LM group compared to those in the LK group, indicating that the mouse livers generated an inflammatory response (Figure 8). NF-κB-p65 expression in the LGTD group exhibited no significant alterations compared with that in the LM group, indicating that the liver injury in the LGTD group was not significantly improved. NF-κB-p65 expression in the LY, LGTZ, and LGTG groups was significantly increased. MyD88 expression in the rest of the dosage groups was significantly decreased compared with that in the LM group in a dose-dependent manner, indicating that the inhibitory effect of *G. lingzhi* triterpenoids on MyD88 expression was more significant with increased dosage and suppressed the inflammatory factors in the liver tissues.

The MyD88/NF-κB-p65 signaling pathway, which stimulates the secretion of inflammatory factors, has been implicated in hepatic pathological alterations and aberrant liver function. *G. lingzhi* triterpenoids attenuated CTC-induced liver injuries. Consequently, our investigation may facilitate future research on the mechanisms by which *G. lingzhi* triterpenoids protect the liver.

## 4. Discussion

*Ganoderma* triterpenoids comprise ganoderic acid A, ganoderic acid D, ganoderic acid C2, ganoderic acid F among other compounds [30]. The triterpenoid composition and content vary across different *Ganoderma* species [31]. Additionally, triterpenoid content varies by *Ganoderma* tissue type, including fruiting body, mycelium, and spore powder [32]. The major triterpenoid components in *G. lingzhi* spores are ganoderic acid A and ganoderic acid G [33]. Therefore, this study conducted quantitative analysis of ganoderic acid A and ganoderic acid G isolated from *G. lingzhi* spore powder. Given the complexity of triterpenoid composition, the observed in vivo effects are likely due to synergistic interactions among multiple components, not solely attributable to ganoderic acids A and G. Comprehensive metabolomic or LC-MS/MS analyses are required to fully identify individual compounds and elucidate their respective roles. The extraction procedure was previously validated in our laboratory [26], with reported RSD values of 1.17% and 1.25% for the two major ganoderic acids, indicating good reproducibility. A single batch was used for all experiments to minimize batch-to-batch variability.

*G. lingzhi* triterpenoids exhibit antioxidant, anti-inflammatory, hypoglycemic, anticarcinogenic, and antitumor properties and have been used to manage and prevent a range of diseases, such as hepatitis and diabetes. Qin et al. demonstrated that *G. lingzhi* triterpenoids are effective in treating Alzheimer’s disease through multiple pathways, including attenuating neuroinflammatory responses, inhibiting apoptosis, and modulating autophagy disorders, thereby exhibiting multi-target and multi-pathway properties [34]. Sudthirak et al. demonstrated that *G. lingzhi* can be used as a functional food to prevent or slow the progression of conditions such as hypercholesterolemia [35]. Furthermore, Wu reported that various *G. lingzhi* triterpenoids exhibit potent antioxidant activity [36].

*G. lingzhi* triterpenoids exhibit significant hepatoprotective effects. Lu et al. reported that *G. lingzhi* and its products demonstrate preventive and therapeutic effects against various types of hepatic injury, including alcoholic liver disease, drug-induced, toxin-induced, viral hepatitis, and ischemia–reperfusion injury [37]. These compounds slow disease progression, suppress stellate cell proliferation, reduce hepatic fibrosis, inhibit hepatocellular carcinoma cell growth and migration, and promote apoptosis in hepatocellular carcinoma cells. Subsequently, studies have investigated the protective effects of *G. lingzhi* extracts, mycelia, and spores against chemical-induced liver injury in mice [38,39,40]. Huang et al. reported that *G. lingzhi* spore oil significantly enhanced cellular immune function and NK cell activity in mice, conferring protective effects against CCl_4_-induced liver injury [41]. Wu et al. demonstrated that *G. lingzhi* triterpenoids upregulate SOD and CAT activities in liver tissues, thereby exerting antioxidant and free radical scavenging effects [42].

In addition to *G. lingzhi*, numerous other plants and their secondary metabolites have been demonstrated to protect against acute liver injury through comparable mechanisms involving antioxidant and anti-inflammatory pathways. Zhang et al. demonstrated that liensinine, an alkaloid derived from *Nelumbo nucifera* (lotus) seed embryos, exerts potent anti-inflammatory and antioxidant effects by inhibiting NF-κB signaling and activating the Nrf2/HO-1 pathway [43]. Nan et al. investigated the effects and underlying mechanisms of *Gynostemma pentaphyllum* saponins against acute alcohol-induced liver injury in mice [44]. Furthermore, treatment with Gynostemma pentaphyllum saponins in a dose-dependent manner significantly attenuated alterations in the aforementioned markers and effectively protected against acute alcohol-induced liver injury in mice by activating the Nrf2/NF-κB signaling pathway. These findings underscore the central role of Nrf2 and NF-κB pathways in hepatoprotection, which is consistent with the mechanism proposed for *G. lingzhi* triterpenoids in the present study.

In this study, we investigated the hepatoprotective mechanism of *G. lingzhi* triterpenoids against carbon tetrachloride-induced acute liver injury in mice. Analysis of liver index revealed that *G. lingzhi* triterpenoids significantly ameliorated hepatic damage induced by carbon tetrachloride. Serum ALT and AST levels indicated that *G. lingzhi* triterpenoids effectively preserved hepatocytes and attenuated hepatic damage. At low doses, *G. lingzhi* triterpenoids demonstrated limited amelioration of acute liver injury, as evidenced by MDA levels and SOD activity, which aligned with the CAT and GSH-Px indices. Consistently, Ao et al. reported that *G. lingzhi* triterpenoids reduced ALT and AST serum levels, decreased hepatic MDA content, enhanced SOD activity, and ameliorated liver injury [45]. IL-6 and TNF-α are key inflammatory cytokines mediating inflammatory reactions, and *G. lingzhi* triterpenoids significantly inhibited these inflammatory factors, thereby reducing hepatocellular injury. This finding aligns with Wang et al., who demonstrated that *G. lingzhi* triterpenoids reduced TNF-α expression in the serum of rats with liver damage [46]. Histopathological analysis revealed that higher doses of *G. lingzhi* triterpenoids correlated with reduced hepatic injury. Li et al. concluded that *G. lingzhi* triterpenoids significantly attenuated hepatic injury in mice, a finding consistent with the results of the current study [47]. The effects of pathways related to liver function in mice were analyzed. The oxidative factor Nrf2 typically inhibited by Keap1, was released upon Keap1 downregulation, resulting in significantly elevated Nrf2 levels. However, *G. lingzhi* triterpenoids treatment elevated the ex-pression levels of both proteins. This phenomenon aligns with Iranshahy et al., who demonstrated oxidative stress in tissues by assessing Keap1 and Nrf2 expression [48]. The MyD88/NF-κB-p65 signaling pathway drives inflammatory factor secretion during liver injury, resulting in pathological changes and functional abnormalities in the liver. Notably, MyD88 expression was suppressed in the medium and high *G. lingzhi* triterpenoids dosage groups, which subsequently suppressed pro-inflammatory factors in the liver tissues, consistent with Kushairi et al., who established a model of acute hepatic damage and examined the expression of proteins related to inflammatory response [49].

In this study, each experimental group consisted of 8 mice. The sample size was determined based on previous publications using the same CCl_4_-induced acute liver injury model, in which 8 animals per group were sufficient to detect significant differences in serum biochemistry, liver histology, and protein expression [50]. No formal power analysis was performed, but the observed effect sizes (e.g., ALT, AST, and Western blotting results) were consistent with those reported in the literature, supporting the adequacy of the sample size. Because the study was exploratory with pre-specified comparisons, no formal adjustment for multiple testing was made. The *p*-values are therefore nominal.

The LD_50_ (lethal dose 50) of *Ganoderma* triterpenoids is >10 g/kg, classifying them as biologically active substances that are effectively non-toxic. In this study, following the principle of dose gradient design in animal experiments (with a low, medium, and high dose ratio of 1:2:4), the doses were set at 150, 300, and 600 mg/kg/day [27]. Converted based on body surface area, the human equivalent doses of the triterpenes for a 60 kg adult are approximately 0.73, 1.46, and 2.92 g/day, respectively. These values fall within the typical daily intake range (0.5–3 g/day) of commercially available *G. lingzhi* products. No adverse reactions were observed. However, caution is warranted when directly extrapolating these results to humans, and the findings of this study are limited to preclinical data.

*G. lingzhi* triterpenoids decreased serum AST and ALT levels in mice with acute hepatic injury. They also protected hepatocytes, reduced the MDA levels, and increased SOD, CAT, and GSH-Px activities in liver tissue. *G. lingzhi* triterpenoids enhanced oxidative stress resistance and suppressed serum TNF-α and IL-6 levels, indicating reduced inflammation in injured liver tissue.

Hematoxylin-eosin staining (HE staining) revealed that *G. lingzhi* triterpenoids improved hepatic histopathology, preserved cell morphology, and reduced inflammatory infiltration. These findings indicate that *G. lingzhi* triterpenoids attenuated liver damage by suppressing inflammation and enhancing antioxidant capacity.

*G. lingzhi* triterpenoids upregulated Keap1 and Nrf2, expression in the antioxidant pathway, demonstrating their regulatory role in responding to oxidative stress. *G. lingzhi* triterpenoids also inhibited inflammatory pathway proteins NF-κB-p65 and MyD88, indicating suppression of hepatic inflammation.

The liver injury, antioxidant, and inflammatory indices, as well as the expression of proteins related to the antioxidant (Keap1-Nrf2) and inflammatory (NF-κB-p65-MyD88) pathways in mouse liver tissues, indicated that *G. lingzhi* triterpenoids have inhibitory and modulatory effects on oxidative stress and inflammatory responses in acute liver injury and protect the hepatic tissues.

Although our results demonstrate a strong correlation between the modulation of Keap1/Nrf2 and MyD88/NF-κB pathways and the hepatoprotective effects of *G. lingzhi* triterpenoids, causality cannot be definitively established from correlative data alone. To further validate the causal roles of these pathways, future studies should employ specific inhibitors (e.g., ML385 to inhibit Nrf2, or BAY 11-7082 to block NF-κB activation) or genetic silencing approaches (e.g., siRNA or shRNA targeting Nrf2 or MyD88) in the same animal model. Such experiments would confirm whether the observed protection is directly mediated through these pathways. Such experiments are beyond the scope of this work. Nevertheless, our findings provide a solid foundation and clear rationale for such mechanistic validation in future work.

## 5. Conclusions

*G. lingzhi* triterpenoids exhibit significant hepatoprotective, anti-inflammatory, and antioxidant effects in a CCl_4_-induced acute liver injury mouse model. Treatment with *G. lingzhi* triterpenoids reduced serum transaminase levels and liver coefficients, ameliorated histopathological damage, decreased lipid peroxidation (MDA), and increased antioxidant enzyme activities (SOD, CAT, GSH-Px). Furthermore, *G. lingzhi* triterpenoids suppressed pro-inflammatory cytokine production (TNF-α, IL-6) and modulate key signaling pathways involved in oxidative stress (Keap1/Nrf2) and inflammation (NF-κB-p65/MyD88). These findings are consistent with and confirm previous studies on the protective effects of *Ganoderma* triterpenoids. Collectively, these findings indicate that the *G. lingzhi* triterpenoid fraction (containing ganoderic acids A and G) is a promising therapeutic agent for acute liver injury and other oxidative stress-related hepatic disorders, functioning as both hepatoprotective and anti-inflammatory compounds. Further studies are needed to evaluate clinical applicability and safety.

## Figures and Tables

**Table 1:** HPLC gradient elution program.

Time (Min)	A%
20	28
45	39
55	60
75	100
95	100
100	28

**Table 2:** Liver index.

Groups	Dosages (mg/kg/d)	Weight (g)	Liver Weight (g)	Liver Index (%)
LK		33.35 ± 2.87	1.23 ± 0.14	3.69 ± 0.09
LM		35.30 ± 1.44	1.46 ± 0.04	4.14 ± 0.19 #
LY	150	38.59 ± 1.36	1.25 ± 0.15	3.24 ± 0.44 *
LGTD	150	35.38 ± 0.87	1.28 ± 0.11	3.61 ± 0.27 *
LGTZ	300	35.10 ± 0.51	1.31 ± 0.05	3.74 ± 0.20
LGTG	600	32.99 ± 0.38	1.17 ± 0.05	3.54 ± 0.12 *

Note: * *p* < 0.05, # *p* < 0.05.

## Data Availability

All data generated or analyzed during this study are included in this published article.

## Abbreviations

The following abbreviations are used in this manuscript:

ALT	Alanine aminotransferase
AST	Aspartate aminotransferase
BaP	Benzo[a]pyrene
CAT	Catalase from *micrococcus lysodeikticus*
CCl_4_	Carbon tetrachloride
DBP	Dibutyl phthalate
ECL	Enhanced chemiluminescence
GSH-Px	Glutathione peroxidase
HE staining	Hematoxylin-eosin staining
HPLC	High performance liquid chromatography
IL-6	Interleukin-6
MDA	Malondialdehyde
MyD88	Myeloid differentiation factor 88
NF-κB	Nuclear factor-κ- gene binding
SOD	Superoxide dismutase
TBST	Tris-buffered saline with tween
TNF-α	Tumor Necrosis Factor-α
TLR/NF-κB	Toll-like receptors nuclear factor-κ- gene
